# Pan-organ transcriptome variation across 21 cancer types

**DOI:** 10.18632/oncotarget.14303

**Published:** 2016-12-27

**Authors:** Wangxiong Hu, Yanmei Yang, Xiaofen Li, Shu Zheng

**Affiliations:** ^1^ Cancer Institute (Key Laboratory of Cancer Prevention and Intervention, China National Ministry of Education), The Second Affiliated Hospital, Zhejiang University School of Medicine, Hangzhou, Zhejiang 310009, China; ^2^ Key Laboratory of Reproductive and Genetics, Ministry of Education, Women's Hospital, Zhejiang University, Hangzhou, Zhejiang 310006, China; ^3^ Research Center for Air Pollution and Health, School of Medicine, Zhejiang University, Hangzhou, Zhejiang 310009, China

**Keywords:** gene expression, organ-specific genes, pan-cancer, weighted correlation network analysis

## Abstract

It is widely accepted that some messenger RNAs are evolutionarily conserved across species, both in sequence and tissue-expression specificity. To date, however, little effort has been made to exploit the transcriptome divergence between cancer and adjacent normal tissue at the pan-organ level. In this work, a transcriptome sequencing dataset from 675 normal-tumor pairs, representing 21 solid organs in The Cancer Genome Atlas, is used to evaluate expression evolution. The results show that in most cancer types, gene expression divergence and organ-specificity are reduced in cancer tissue compared to adjacent normal tissue. Furthermore, we observe that all cancers share cell cycle dysregulation through interrogating differentially expressed protein coding genes. Meanwhile, weighted correlation network analysis is used to detect of the gene module structure variation between cancer and adjacent normal tissue. And modules consisting of tightly co-regulated genes in cancer change substantially compared with those in adjacent normal tissue. We thus assume that the destruction of a coordinated regulatory network might result in tumorigenesis and tumor progression. Our results provide new insights into the complex cancer biology and shed light on the mysterious regulation mode for cancer.

## INTRODUCTION

Cellular phenotype and organ formation are largely shaped by dynamic transcriptional regulation [1, 2]. Gene expression profile variation has an essential role in understanding the fundamental molecular events in human biology and transition to disease. Son et al. [3] explored the genome-wide expression profiling of 19 normal tissues in 30 individuals using microarrays and revealed that the expression profiles belonging to the same organ clustered together. More recently, Pervouchine et al. [4] characterized the transcriptional profiles of a large, heterogeneous collection of murine tissues by RNA sequencing and identified a distinct core set of genes that were involved in basic functional and structural housekeeping processes common to all cell types. They proposed that perturbation of these conserved genes was associated with embryonic lethality and cancer. Gene expression profiling is also widely used in tumor molecular typing [5, 6] and prediction of recurrence [7, 8] and survival [9–11].

Nevertheless, systematical pan-organ and population- based transcriptome analysis may be hampered by a lack of sufficiently related datasets prior to the Cancer Genome Atlas (TCGA) [12] and Genotype-Tissue Expression (GTEx) project [13, 14]. In addition to normal tissue transcriptome data in a GTEx project, a matched tumor transcriptome dataset is available from TCGA, which provides a good opportunity for elucidating the transcriptional variation between normal and tumor tissues and the underlying genetic basis of normal → tumor transition. Previously Kaczkowski et al. [15] used TCGA data to identify differentially expressed genes (DEGs) in 14 solid cancer types. However, they used all tumor samples (while the majority of tumors have no matched normal tissue RNAseq dataset in TCGA) instead of choosing matched normal-tumor pairs, and the results may be biased by the tumor heterogeneity. Furthermore, they did not detect gene co-regulation modules in either normal or tumor tissue.

Here, we comprehensively analyze the TCGA solid tissue data, including RNA sequencing of 1,350 matched normal and tumor samples from 675 individuals, representing 21 solid organs ((bladder urothelial carcinoma (BLCA), breast invasive carcinoma (BRCA), cervical squamous cell carcinoma and endocervical adenocarcinoma (CESC), cholangiocarcinoma (CHOL), colorectal cancer (CRC, colon adenocarcinoma (COAD)/rectum adenocarcinoma (READ)), esophageal carcinoma (ESCA), head and neck squamous cell carcinoma (HNSC), kidney chromophobe (KICH), kidney renal clear cell carcinoma (KIRC), kidney renal papillary cell carcinoma (KIRP), liver hepatocellular carcinoma (LIHC), lung adenocarcinoma (LUAD), lung squamous cell carcinoma (LUSC), pancreatic adenocarcinoma (PAAD), prostate adenocarcinoma (PRAD), skin cutaneous melanoma (SKCM), stomach adenocarcinoma (STAD), thyroid carcinoma (THCA), thymoma (THYM), and uterine corpus endometrial carcinoma (UCEC)). For simplicity, acronyms suffixed with N and T indicated normal tissue and the corresponding tumor, respectively (BRCA_N indicates normal breast tissue and BRCA_T indicates breast cancer). The results show that expression divergence (1-*ω* (pairwise Pearson's correlation coefficient)) is significantly reduced in 11 cancer types than in corresponding normal tissues. Further comparison of tumor and adjacent normal tissue samples reveal that all cancers share cell cycle dysregulation. In the meantime, we use weighted correlation network analysis (WGCNA) to detect gene module structure variation between cancer and adjacent normal tissue. It is interesting to note that the sets of tightly co-regulated gene modules in normal tissue are changed in cancer. Our results provide important insights into individual transcriptional variation and the molecular regulation mechanism of the normal tissue → tumor transition.

## RESULTS

### Global patterns of tissue expression

**Figure 1:**
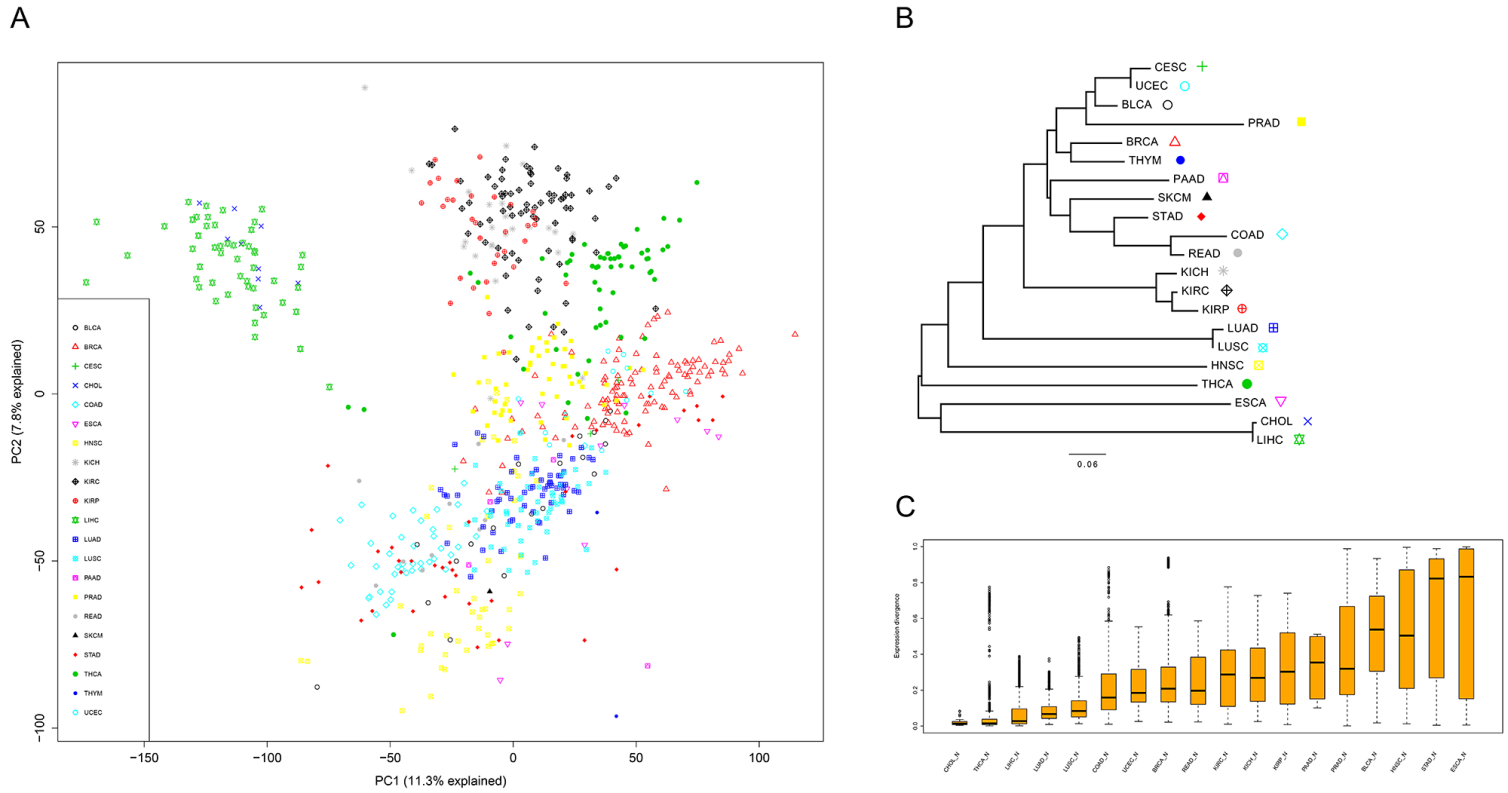
The transcriptome across 21 solid tissues **A.** Sample and normal tissues with similarity based on PCA. **B**. Unrooted NJ tree to infer the evolutionary distances of tissue expression. The tree branch length represented the degree of tissue expression divergence. **C**. Expression divergence of the 18 tissues computed based on the pairwise Pearson's correlation coefficient of the tissue expression profiles.

The RNAseqV2 Level3 data of the 21 tissues were downloaded from TCGA (October 2015). The data set was compiled from 675 matched pairs of tumor and adjacent normal tissues (BLCA-19, BRCA-113, CESC-3, CHOL-9, CRC-32 (COAD-26 and READ-6), ESCA-11, HNSC-43, KICH-25, KIRC-72, KIRP-32, LIHC-50, LUAD-58, LUSC-51, PAAD-4, PRAD-52, SKCM-1, STAD-32, THCA-59, THYM-2, and UCEC-7; see Supplementary Table 1 for more detail). To explore the primary expression pattern in these tissues, we performed a principal component analysis (PCA) on the compiled normal tissue and matched tumor data set (Figure 1A). Samples were grouped together according to tissue types (Figure 1A). As expected, tissues belonging to homologous organs (e.g., COAD and READ, LUAD and LUSC, KICH, KIRC, and KIRP) were distinctly grouped together, suggesting they have the same embryonal origin. Notably, LIHC and CHOL were mixed together and were relatively far from the rest of tissues. This further strengthens the notion that tissue originating from the same germ layer harbors a similar expression pattern. To further explain the divergence of tissue expression, we constructed a genealogy of tissues using a neighbor-joining (NJ) algorithm based on the centroid expression of the median expression across all samples of a given tissue (Figure 1B). The distance matrix used in the NJ method was derived as 1-*ω*, where *ω* is the pairwise Pearson's correlation coefficient of the tissue expression profiles (Figure 1C). The NJ method generated a tree whose total branch length should be the smallest of the observed pairwise distances. In other words, the branch length summarized the expression divergence of different tissues; longer branches (both internal and terminal horizontal branches) imply higher levels of tissue expression divergence. Notably, tissues belonging to homologous organs were closely clustered together and harbored shorter branches (Figure 1B), which was in accordance with the PCA results.

Furthermore, to quantify the expression divergence of samples in each tissue, we calculated the pairwise Pearson's correlation coefficient (*ω*) of the samples. Then, 1-*ω* was used to estimate the divergence across samples. CHOL and THCA exhibited minimum divergence (< 0.1) compared with other tissues (Figure 1C). In contrast, the median divergence exceeds 0.5 in four tissues, BLCA, HNSC, STAD, and ESCA, suggesting high gene expression diversity is present in these tissues.

### Convergent expression patterns in tumors

**Figure 2:**
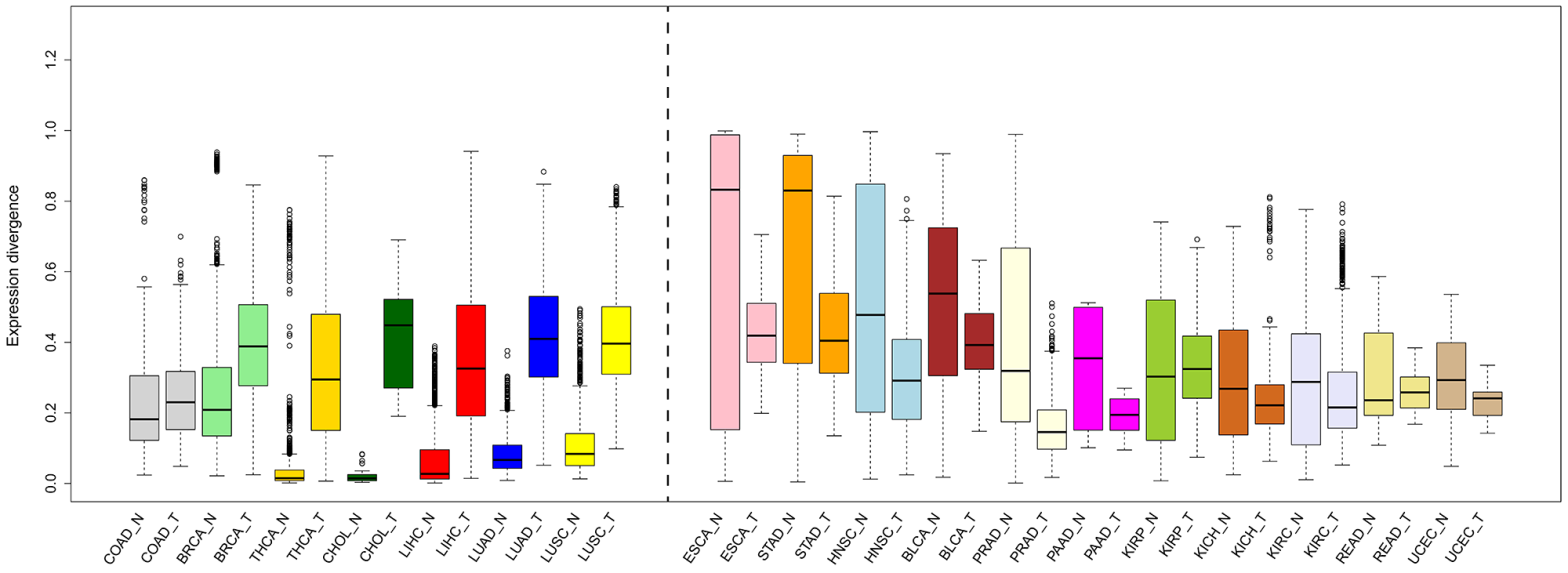
Comparison of expression divergence between normal tissues and their matched tumors The median values (black line in the box) are indicated.

Comparison of global expression divergence between matched tumors and adjacent normal tissues revealed clear differences, except in the case of COAD. In short, two patterns, enhanced expression divergence (BRCA, CHOL, LIHC, LUAD, LUSC, and THCA) and reduced expression divergence (BLCA, ESCA, HNSC, KICH, KIRC, KIRP, PAAD, PRAD, READ, STAD, and UCEC), were observed in cancer (Figure 2). Of special interest is the inquiry of the PCA and mode of evolution of mRNA expression, and we found an overall reduced divergence between tumors (Supplementary Figure 1A), indicating that the transcriptome of different cancers converged to a similar mode. Likewise, the branches along the tumor NJ tree shortened (Supplementary Figure 1B). Additionally, the topology was largely reshaped in cancer (Figure 1B and Supplementary Figure 1B). We thus combine the normal and cancer data to reconstruct the NJ tree. Surprisingly, some cancers, such as BLCA, BRCA, CESC, LUAD, and LUSC, were mixed and separated from their respective normal tissue, exceeding the boundary of tissue-specificity (Supplementary Figure 2).

### Organ-specific genes were weakened in tumors

**Figure 3:**
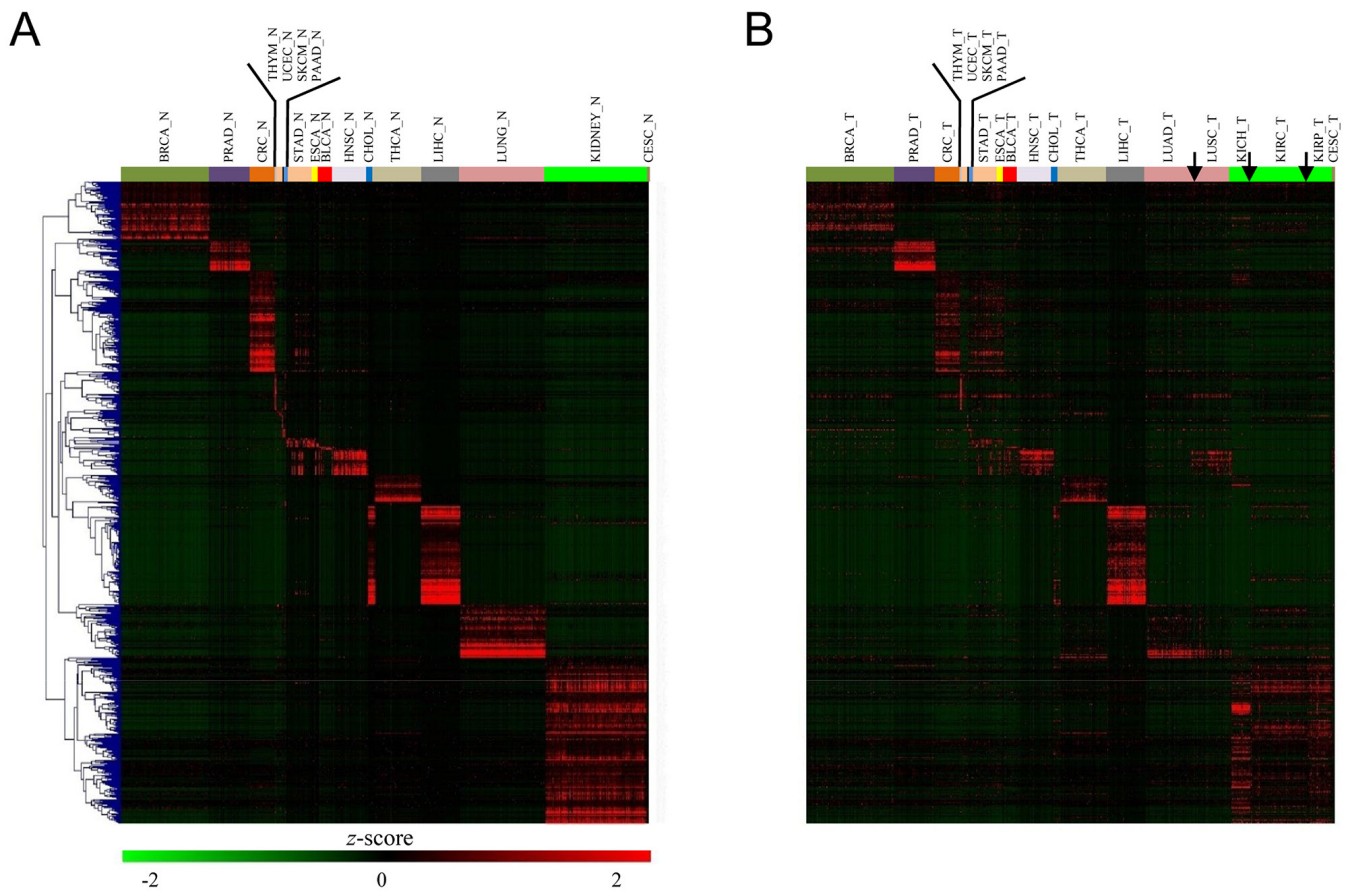
Organ-specific gene expression variation between normal and tumor tissues **A**. Heatmap of the expression level of all 675 normal samples across 21 tissues for the top 821 organ-specific genes. **B**. Heatmap of the expression level of all 821 organ-specific genes in tumors ordered by hierarchical clustering in (A). Notably, significantly lower expression levels for these organ-specific genes were observed in tumors.

As noted earlier, some cancers were mixed together and broke the rule of tissue-specificity. Then, it is tempting to speculate that the organ-specific genes should be weakened in tumors. To this end, we identified the genes that were specifically over-expressed in one organ compared all other 20 organs. We found that kidney, liver, cholangio, colorectum, breast, lung, thyroid, and prostate tissue harbored the largest number of specifically expressed genes (Figure 3A). For instance, we found breast-specific genes enriched in lactation and mammary gland development, prostate-specific genes enriched in prostate gland morphogenesis and development, thyroid-specific genes enriched in thyroid hormone generation and thyroid gland development, and lung-specific genes enriched in respiratory gaseous exchange and immune response. In this context, these genes, in most cases, reflected organ-specific functions. Specifically, cholangio and liver tissues globally share similar expression profiles and we simultaneously selected their organ-specific genes. Then, we investigated the expression pattern of organ-specific genes in tumors. Most organ-specific genes were significantly weakened (*P* < 0.001 by paired *t*-test) in tumors, except for in the prostate, liver, and thymus. Especially for cholangio, almost all organ-specific genes disappeared, which is in sharp contrast with the basically unaltered state in the liver (Figure 3B). Moreover, colorectum-specifically expressed genes, such as carcinoembryonic antigen-related cell adhesion molecule 5 (*CEACAM5*), lectin, galactoside-binding, soluble, 4 (*LGALS4*), mucin 13 (*MUC13*), and calpain 5 (*CAPN5*), were moderately expressed in both CRC and STAD, suggesting that a group of organ-specific genes disobeyed tissue-specificity (or, more accurately, tumor-specificity).

### Identification of differentially expressed genes in different cancers

**Figure 4:**
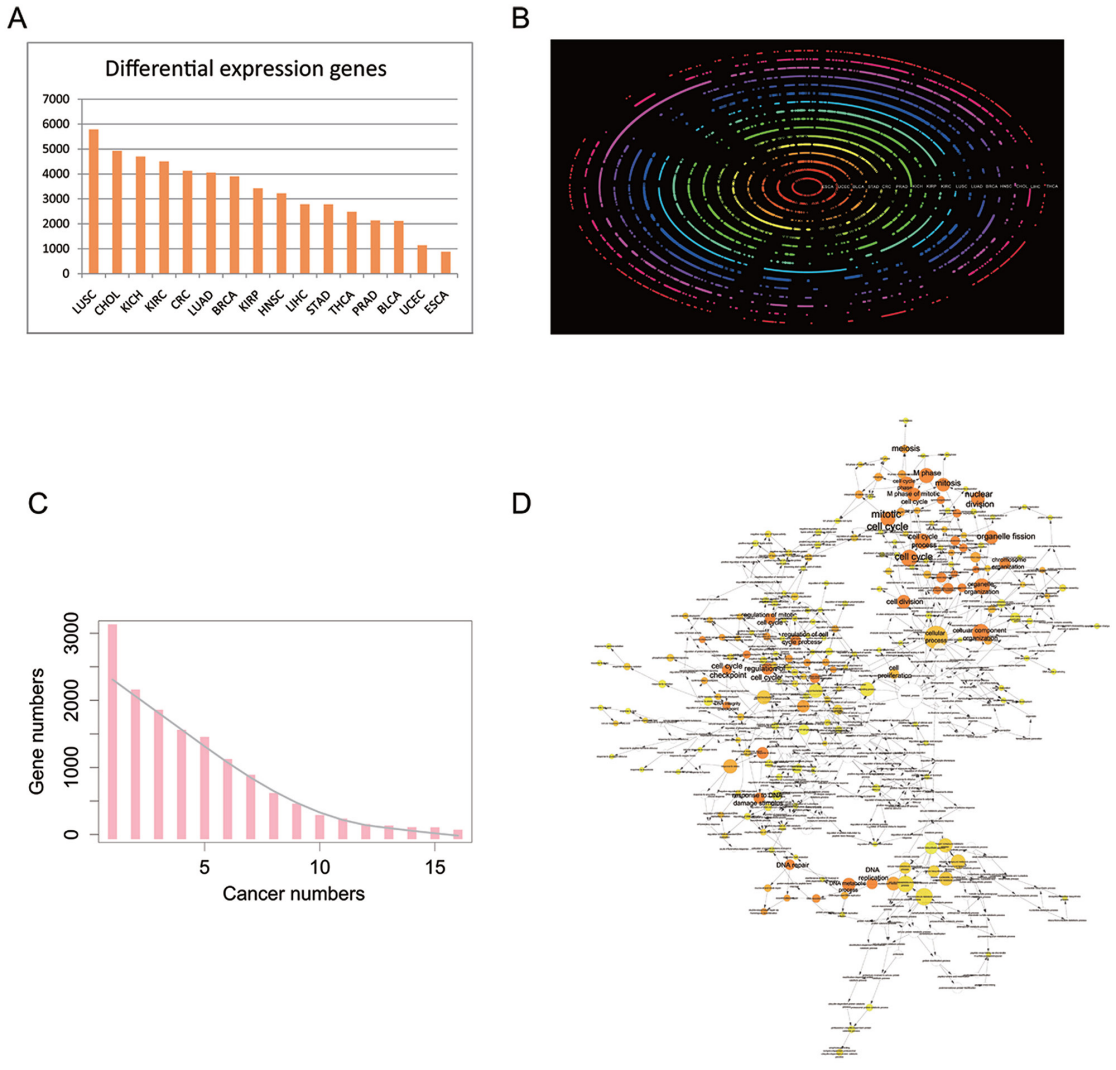
Differential expression protein coding genes across 16 tissue types **A**. The number of DEGs in 16 tissue types. **B**. Bi-clustering of the DEGs across 16 tissue types and transformed into polar coordinates for better visualization. The colored concentric circles represent the DEGs in each tissue. **C**. The number of DEGs across tissues. The grey smooth line was fitted by lowess method. **D**. GO enrichment for DEGs in at least 12 cancer types under the Biological Process term. The node size is proportional to the number of genes in the GO category. The color corresponds to the enrichment significance, and a deeper color indicates higher enrichment significance. As for white nodes, they are not enriched, but only display the hierarchical relationship among these ontology branches.

It is widely accepted that genes dysregulated in different cancer types are clinically attractive as diagnostic biomarkers or therapeutic targets. To this end, it is critical to determine the shared combination of common driver genes across different cancers. In view of the tumor heterogeneity, we only considered matched tumor samples for differential expression analysis because the number of tumor samples is much larger than that of normal samples in TCGA. Furthermore, four cancers (CESC, PAAD, SKCM, and THYM) were excluded because their sample sizes were too small (less than eight) to achieve powerful statistical significance. Additionally, COAD and READ were combined into CRC according to the traditional classification. The number of DEGs ranged widely from 876 in ESCA to 5,788 in LUSC (Figure 4A, all DEGs were listed in Supplementary Table 2), with a median of 3,323, suggesting that the genes that are dysregulated in different cancers are tissue-specific. Notably, aberrant expression of five cancer-related genes alcohol dehydrogenase 1B (*ADH1B*), mitotic checkpoint serine/threonine kinase B (*BUB1B*), cell division cycle 45 (*CDC45*), FXYD domain containing ion transport regulator 1 (*FXYD1*), and kinesin family member 20A (*KIF20A*) were observed in all 16 cancers. *ADH1B* was markedly repressed in all the 16 tumors (average fold change (FC): 0.02) and *FXYD1* was down-regulated in almost all tumors (average FC: 0.13), except for KIRC (up-regulated, FC: 2.33). In contrast, *BUB1B*, *CDC45*, and *KIF20A* were highly expressed in all 16 tumors with an average FC at 10.04, 12.42, and 9.83, respectively.

Meanwhile, gene ontology (GO) analysis of DEGs (244 genes) in at least 12 cancers revealed that they were mainly enriched in the cell cycle (FDR corrected *P*-value 1.6260E-40, 77 genes), organelle organization (FDR corrected *P*-value 5.6591E-18, 68 genes), mitosis (FDR corrected *P*-value 1.3085E-35, 45 genes), etc., which were all closely related to tumor characteristics (Figures 4B, , and 4D). Therefore, widespread differential expression of the activators (e.g., CDC families, CCNF, and MKI67) suggests their crucial roles in tumorigenesis and development.

Additionally, we selected 1,584 genes that are differentially expressed in over half of 16 cancer types and sought survival-related DEGs in nine cancers (including BLCA, CRC, ESCA, HNSC, KIRC, KIRP, LIHC, LUAD, and UCEC). The patients for nine cancers were divided into two groups with high and low prognostic index (PI, see methods for more detail) in each cancer. Additionally, they can be significantly separated into two groups (Supplementary Figure 3, logrank test, *P* < 0.05) based on 3~25 survival-related DEGs in each cancer (Supplementary Table 3). These survival-related DEGs can be prognostic signatures in cancers, but they warrant further validation.

### Altered mRNA co-regulation modules in tumors

**Figure 5:**
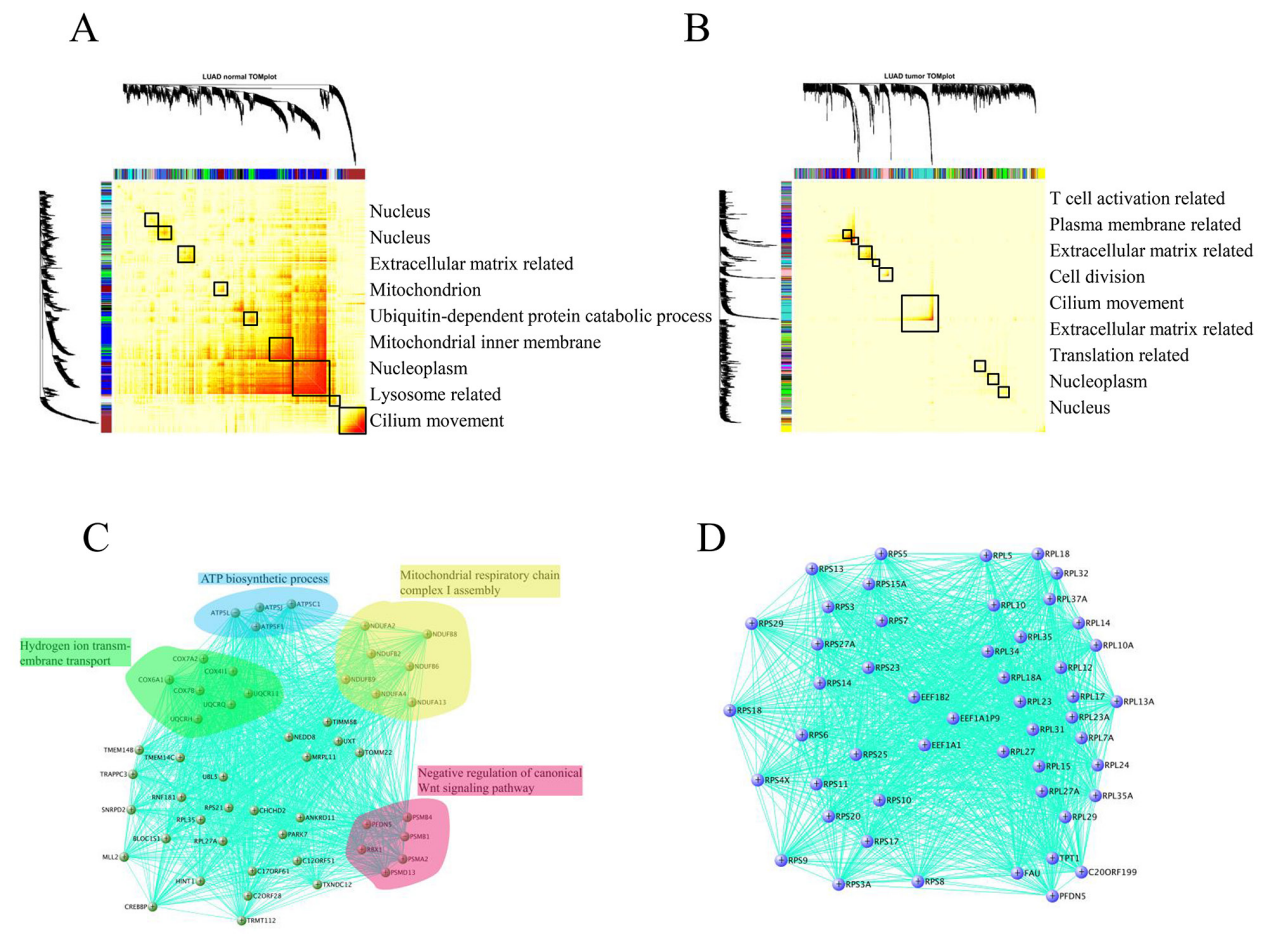
Comparison of the module structure in normal lung tissue A. and lung adenocarcinoma B. using WGCNA To group genes with high topological overlap into modules (also known as clusters), average linkage hierarchical clustering coupled with the TOM distance measure were used. Once a dendrogram was obtained from hierarchical clustering, we selected a height cutoff to achieve clustering. Here, modules corresponded to dendrogram branches. Rows and columns corresponded to genes. Black boxes along the diagonal were modules and color bands corresponded to modules, with their GO annotation listed in right pannel. Evidently the gene modules (sets of tightly co-regulated genes) in normal tissue were nearly reshaped in matched tumors. **C**. Modules related to the fundamental respiratory system in lung tissue was lost in LUAD. **D**. A peculiar sub-network comprised of ribosomal proteins was observed in LUAD. For better readability, we only kept the 50 top hub genes in the module.

As described above, the expression divergence was mitigated and cell cycle process was disturbed in many cancers relative to their adjacent normal tissues. To further elucidate the expression variation between normal and matched tumors, we sought co-expressed gene modules using weighted correlation network analysis (WGCNA). This powerful tool can determine the core gene regulatory modules in different tissues that can adapt their molecular functions to specialized roles (e.g., tissue-specificity) and shed light on the intrinsic expression variation between different datasets (Herein: normal vs. cancer) in terms of RNA-Seq data [16]. Intriguingly, modules consisting of sets of tightly co-regulated genes unveiled by a topological overlap matrix plot (TOMplot) differed considerably between normal and cancer tissues (Figure 5A and 5B). In CRC, KIRP, LUSC, and THCA, the module structure was altered between normal and cancer tissues (Supplementary Figure 4). Additionally, in BRCA, LIHC, and PRAD, more modules were found in cancers than in normal tissues. Last but not the least, it is of primary interest to identify co-expressed gene modules that were diminished or lost in HNSC, KICH, KIRC, LUAD, and STAD, which is in sharp contrast with the case for BRCA (Supplementary Figure 4). Further exploration of sub-networks in lung tissue showed that the basic respiratory function was lost in LUAD (Figure 5C). Surprisingly, in LUAD, a sub-network comprised of ribosomal proteins was observed (Figure 5D). Therefore, it is not surprising that the concerted gene expression regulatory networks are destroyed in cancer.

## DISCUSSION

The evolution of gene expression pattern has become a subject of increasing interest for diversity in scientific research during the past few years [17–21]. Recently, Gerstein et al. [19] used the comparative genome method to explore the transcriptome across distant species (humans, worms, and flies) and discovered these animals share co-expression modules, many of which were enriched in developmental genes. Resembling the findings of Gerstein et al., Berens et al. [22] compared the transcriptome in Hymenoptera (bees, ants, and wasps) and unveiled significant overlap in metabolic pathways and gene functions associated with the convergent evolution of castes, especially those related to carbohydrate and amino acid metabolism, morphogenesis, oxidation–reduction, and transcriptional regulation. Inspired by the findings of Gerstein et al. [19] and Berens et al. [22] that gene co-regulation modules are conservatively present in distant species during long-term evolution, we studied the expression divergence between 21 normal organs and corresponding tumors in 675 individuals. Two thirds of cancer types showed reduced divergence compared to normal tissues, suggesting that tumors converge to an unknown stable state. Further identification of shared DEGs between normal and tumor tissues uncovers dysregulation of cell cycle processes, which is one of the hallmarks in cancer [23]. We postulated that the robust regulatory network orchestrated by cell cycle related genes would be impaired or altered if they were differentially expressed.

Of particular concern in this work is that the expression levels of organ-specific genes are decreased or not present in many tumor types, which is similar to tissue dedifferentiation in plants. In cancer, however, the concept that differentiated cells become dedifferentiated has been controversial. Cancer dedifferentiation should uniquely apply to a situation in which a more specialized tissue cell type loses the expression of organ-specific genes related to specialized tissue function [24]. Indeed, in most cancer types, our results corroborate this idea and offer sufficient data supporting the existence of this process. For example, *TPO* is one of the high-ranking genes in thyroid tissue that is significantly down-regulated in THCA. It encodes thyroid peroxidase enzyme, which is a thyroid-specific glycosylated hemoprotein, and aberrant regulation of *TPO* can result in thyroid dyshormonogenesis [25]. Surfactant protein A1 (*SFTPA1*), *SFTPA2*, *SFTPB*, *SFTPC*, and *SFTPD*, which are all related to respiratory gaseous exchange, are the top-ranking lung-specific genes that are remarkably weakened in LUAD and LUSC; however, the roles they play in lung tumors require further elucidation. One of the kidney-specific genes, *KCNJ1* (potassium channel, inwardly rectifying subfamily J, member 1), is required for maintaining potassium balance, which has recently been shown to have low-expression in tumor proliferation and metastasis. Additionally, it is an independent prognostic factor in clear cell renal cell carcinoma [26], which is in line with the observations in this study. In summary, the variety of the organ-specific genes in each organ is closely associated with organogenesis and/or organ-specific functions. In view of this evidence, we firmly believe that the transition to tumorigenesis, progression, or even metastasis, requires relief of modulation from the organ-specific genes.

Next, we compare the co-regulation modules between normal and tumor tissues by WGCNA because perturbation of the cell cycle process and loss of organ-specific gene expression may destroy the tightly regulated network. In contrast to some studies that only use DEGs [27] or top varying genes in the dataset [28], we keep all genes that have a RSEM-normalized count of more than 1 in more than 90% of the samples for each tissue or cancer sample because we focus on the identification of global differences between normal and tumor tissues, which roughly leads to retention of 15,000~16,000 genes. Meanwhile, filtering genes by differential expression is also not recommended by the author of WGCNA because it completely invalidates the scale-free topology assumption and will result in a set of highly correlated genes that will essentially form a single or few correlated modules [29]. As mentioned above, our result showed that the module structure was reshaped in CRC, KIRP, LUSC, and THCA. We reasoned that the dispersed module is prone to evolve novel functions by recruiting new regulatory elements because the newly interacted gene complex should commonly gain subfunctionalization or neofunctionalization before fixation. Once fixed in cancer, newly formed gene regulatory modules may suffer from a different selection pressure to maintain their existence. One of the most promising uses of co-regulation modules is in the exploration of module structures among different cancer TNM stages and study of the correlation between distinctive gene co-expression modules and clinical phenotype diversity because modules that are associated with cancer have been unmasked in breast cancer [30], lung cancer [28], prostate cancer [31], and endometrial cancer [27]. Yang et al. [32] unveil a new perspective that prognostic genes tend to be enriched in the modules that are conserved across four cancer types (GBM, OV, BRCA, and KIRC). However, in this study, we cannot find authentic KIRC modules (Supplementary Figure 4J). One explanation is the rare overlap of samples, and another one can be ascribed to platform discrepancy (their result is based on Agilent 244 K microarray data). Briefly, our data revealed that all cancers shared common module alternations, increase, transform, subside or disappear; namely, each cancer harbored a distinct gene expression pattern from its corresponding normal tissue, but the biological significance in caner requires further elucidation.

Collectively, our results, in combination with previous studies, uncover the basic molecular events occurring during tumorigenesis that appear to be conserved despite the vast differences in origination and physiological features from diverse cancer types. Additionally, all three notable features, cell cycle dysregulation, organ-specific genes weakening, and co-regulatory network reshuffling, can easily distinguish tumor from normal tissue. We believe that the typical characteristics inferred from expression divergence allow us to better understand tumorigenesis, progression, and metastasis.

## MATERIALS AND METHODS

### Gene expression data processing and normalization

All normal and matched tumor level 3 mRNA expression data sets were obtained from the TCGA (October 2015). To obtain high-confidence result, we only considered HiSeq samples for mRNA (RNASeqV2). Batch effects were corrected using the ComBat function implemented in the Bioconductor sva package [33]. RSEM-normalized data for mRNA were log_2_-transformed. The expression values for each gene were further converted to *z*-scores by subtracting the mean and dividing by the standard deviation across each sample. principal component analysis (PCA) on gene expression was performed by function ‘prcomp’ in the ‘stats’ package implemented in R.

### Statistical analysis

Differentially expressed mRNA analysis between normal and tumor tissues was performed by DEGSeq package for R/Bioconductor [34]. Genes with expression level < 1 (RSEM-normalized counts) in more than 50% of samples were removed. Significantly differentially expressed mRNAs were selected according to the false discovery rate (FDR) adjusted *P*-value <0.05 and fold change > 2 condition. Generally, we only considered the tissues with six or more samples for differential expression analysis, which retained 16 pairs of normal tissues and their matched tumors (BLCA-19 pairs, BRCA-113, CHOL-9, CRC-32, ESCA-11, HNSC-43, KICH-25, KIRC-72, KIRP-32, LIHC-50, LUAD-58, LUSC-51, PRAD-52, STAD-32, THCA-59, and UCEC-7). Clustering of DEGs across tissues was performed by seriation package for R and transformed into polar coordinates for better visualization. Then, DEGs across more than 12 cancers were subjected to GO interrogation. The *P*-value was determined by the hypergeometric test with the whole annotation as reference set and then adjusted for multiple testing using the Benjamini-Hochberg FDR correction method. GO enrichment analysis was conducted by BiNGO implemented in Cytoscape [35, 36].

Genes correlated with the patient survival time in multivariate Cox regression analysis were determined using the least absolute shrinkage and selection operator (LASSO) method. The best λ was determined by 10-fold cross-validation using the glmnet package built-in function cv.glmnet [37]. For each cancer, we divided the patients into high- and low-risk groups by calculating the prognostic index (PI) as follows:

$$PI_{k}=\sum_{g=1}^{n}\beta_{g}m_{gk}$$

where *n* is the number of survival correlated genes, *β_g_* is the regression coefficient of the Cox proportional hazard model for gene *g*, and *m_gk_* is the expression level of gene *g* in patient *k*. Patients were then divided into high- and low-risk groups based on the median PI. The survival difference between two groups (good- and bad-prognosis) was tested by the Kaplan-Meier method and analyzed with the log-rank test with functions survfit and survdiff as implemented in the survival package for R [38]. *P* values < 0.05 were considered significant.

### Network analysis of protein coding genes

Matched normal-tumor samples were retained for further analysis. The TOMplot was plotted by WGCNA package for R [29]. To obtain a high-confidence network, we selected tissues and tumors that have at least 20 samples owning to correlations on fewer than 15 samples will be too noisy and affect the network stability. Furthermore, genes whose counts are consistently low (i.e., genes with a RSEM-normalized count of less than 1 in more than 90% of the samples of each tissue or cancer) were removed because low-expressed features tend to reflect noise and correlations based on low counts are not meaningful. Then, the values of the approximately 15,000~16,000 retained genes were added by 1 (to avoid zero) and then log_2_-transformed to perform WGCNA analysis. Briefly, all retained log_2_-transformed genes (nodes) were used to cluster samples and determine if there are any obvious outliers. If so, we choose a height cut and use a branch cut at that height to remove the offending sample(s). Then to construct a weighted gene network, an optimized soft threshold power, *β*, which is the key parameter for warranting both scale-free topology (R^2^ > 0.9) and sufficient node connectivity, was selected to calculate adjacency. To minimize the effects of noise and spurious associations, the adjacency was transformed into a topological overlap matrix (TOM) to calculate the corresponding dissimilarity (1-TOM). Clusters of coexpressed genes were identified by the average linkage hierarchical clustering function *hclust* implemented in WGCNA. Next, to classify genes with coherent expression profiles into modules, the dynamic tree cut method was used for module identification with the minimum size (genes) cutoff at 30. And modules were merged if their correlation exceeded 0.75 (namely, their genes are highly co-expressed) since dynamic tree cut may identify modules whose expression profiles were very similar. Finally, the weighted gene network was visualized by heatmap; each row and column of the heatmap corresponded to a single gene. The heatmap depicted gene adjacencies or topological overlaps with light colors indicating low adjacency (overlap) and darker colors indicating higher adjacency. Moreover, gene dendrograms and corresponding module colors were plotted along the top and left side of the heatmap. Sub-networks constituted by the 50 top hub genes in specific module was visualized by VisANT [39]. All statistical analysis and graphical representations were performed in the R programming language (×64, version 3.0.2) unless otherwise specified.

## SUPPLEMENTARY MATERIALS FIGURES AND TABLES








