# Supplementary material for: Pan-organ transcriptome variation across 21 cancer types
Source: Oncotarget. 2016 Dec 27;8(4):6809–18. doi: 10.18632/oncotarget.14303 (PMC5351671; doi:10.18632/oncotarget.14303)
Supplement: Supplementary file 1 [file oncotarget-08-6809-s001.pdf]

Pan-organ transcriptome variation across 21 cancer types

SUPPLEMENTARY FIGURES AND TABLES

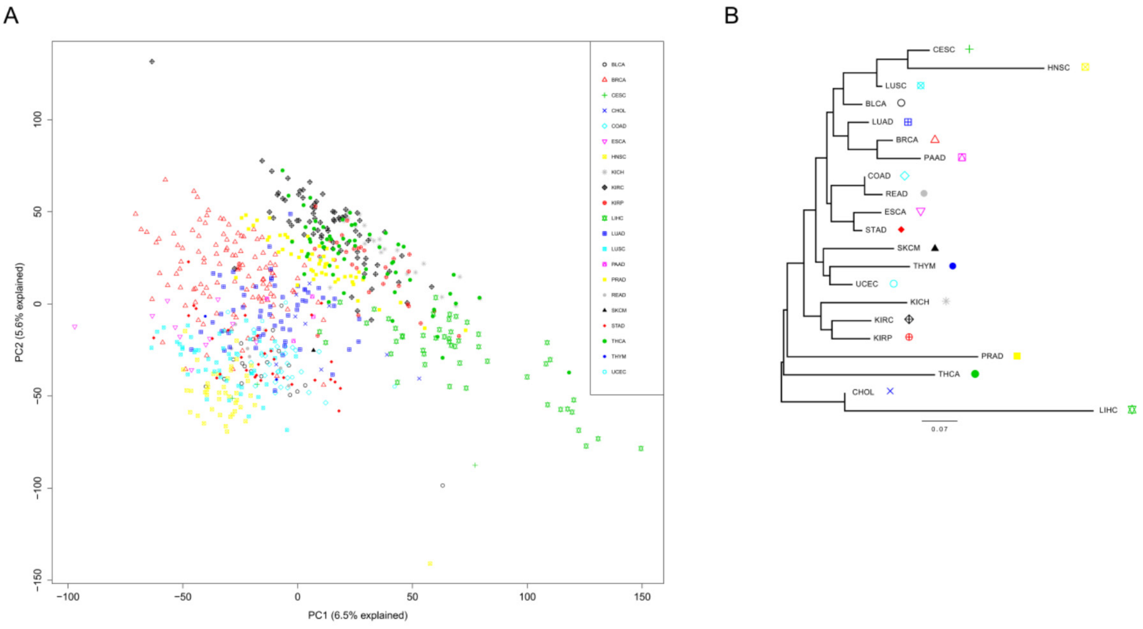

Supplementary Figure 1: The transcriptome across 21 tumor types. A. Visualization of tumor expression similarity based on PCA. **B.** Unrooted NJ tree to infer the evolutionary distances of tumor expression. The tree branch length represented the degree of expression divergence.

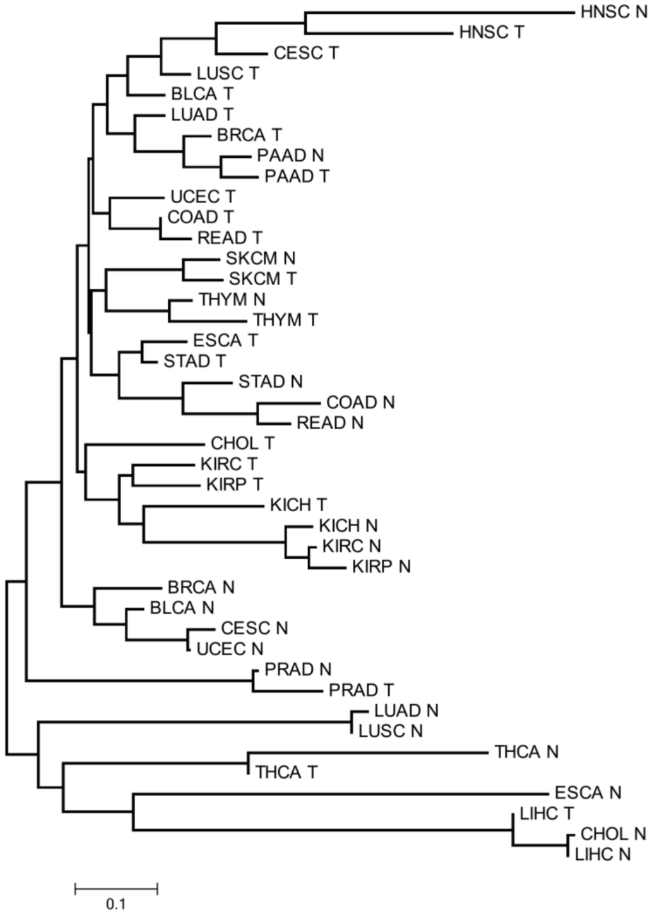

Supplementary Figure 2: Unrooted NJ tree to infer the evolutionary divergence of tumor expression from normal tissue. The tree branch length represented the degree of expression divergence. Of note, in some cases, tissue and corresponding tumor clustered together, suggesting tissue-specificity play a dominant role in these cancers. While in BLCA, BRCA, CESC CHOL, CRC, ESCA, LUAD, LUSC, and UCEC, tumors tend to cluster together rather than by tissue-specificity.

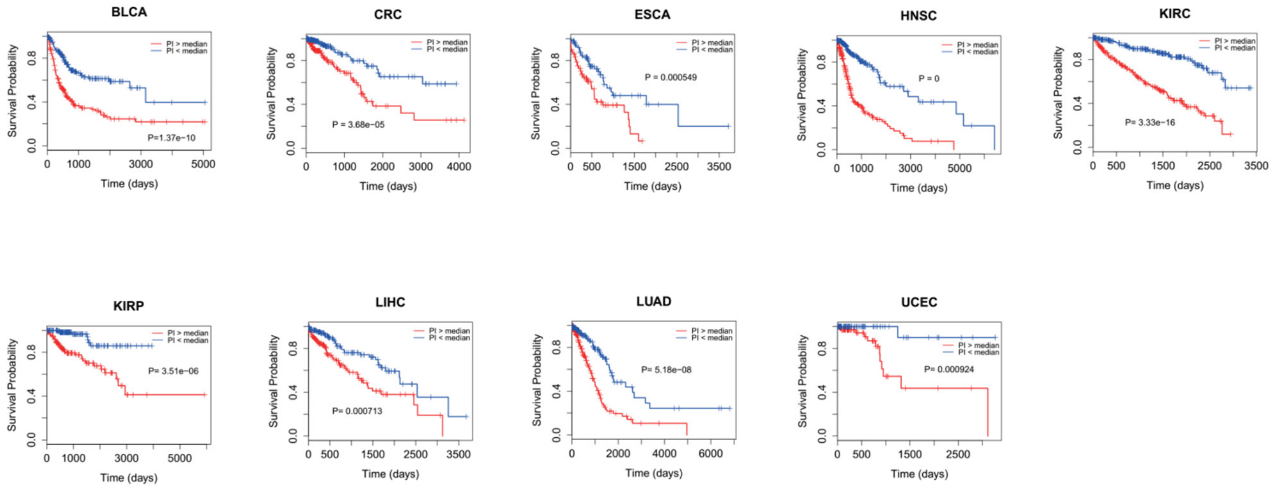

Supplementary Figure 3: Survival analysis of nine cancer types. Kaplan–Meier survival curves for patients defined by high-risk (red curve) and low-risk (blue curve) of overall survival. Statistical difference in clinical outcome is estimated by log-rank test. '+' represents for the censoring samples.

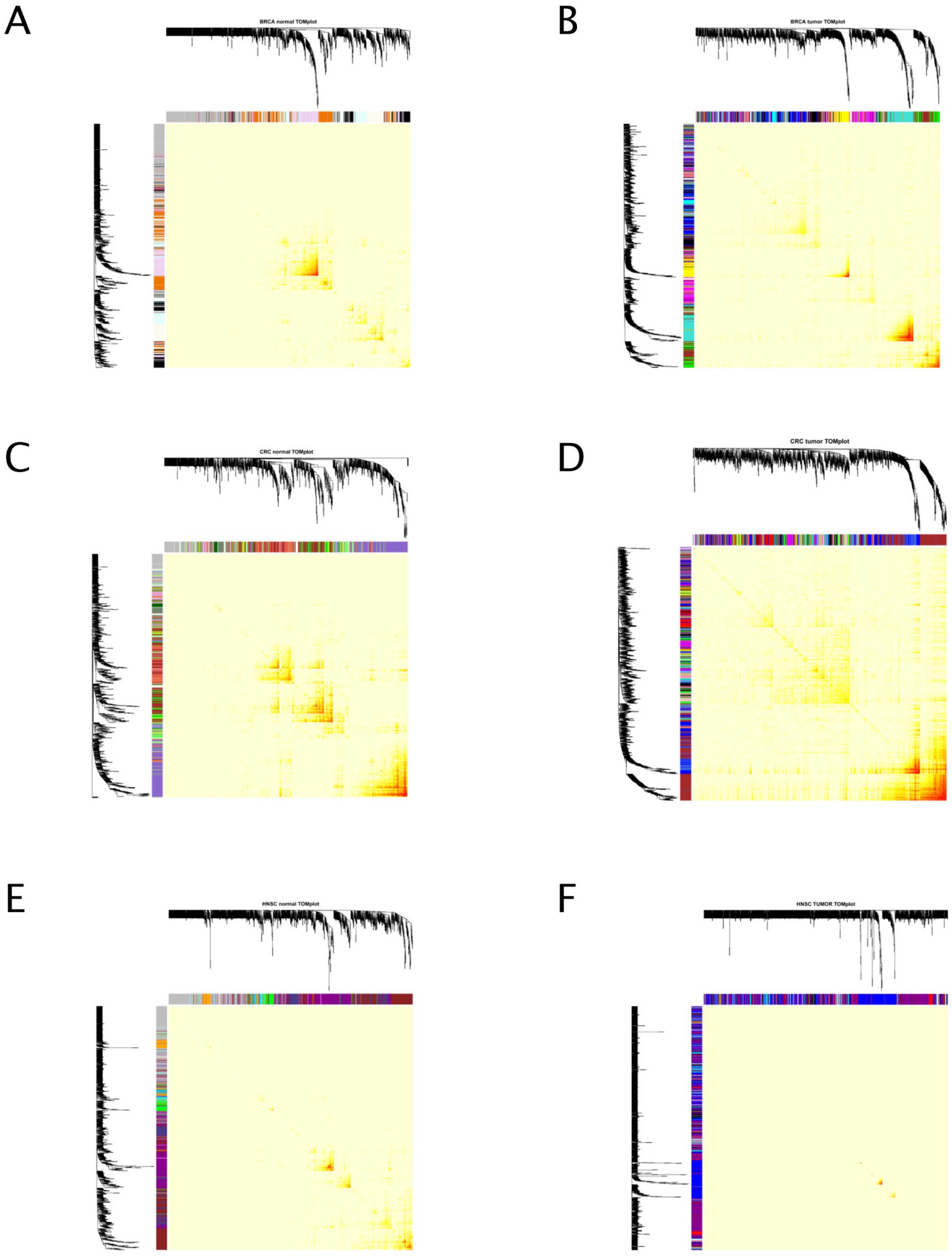
A
BRCA normal TOMplot
B
BRCA tumor TOMplot
C
CRC normal TOMplot
D
CRC tumor TOMplot
E
HNSC normal TOMplot
F
HNSC TUMOR TOMplot

(Continued)

G

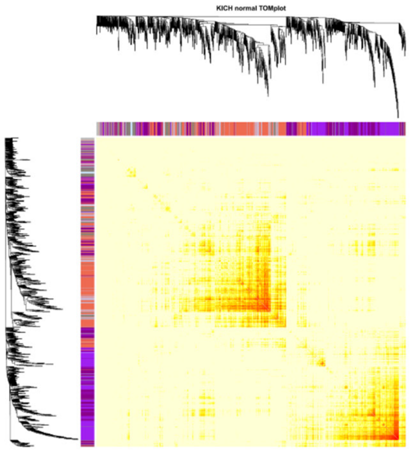

H

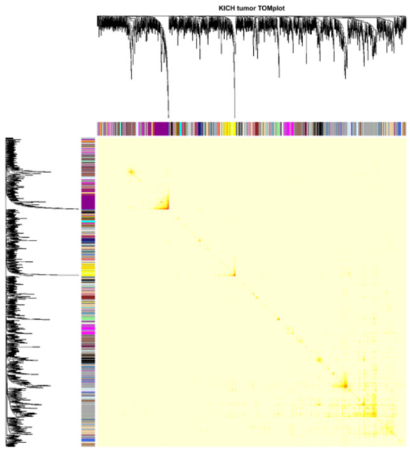

I

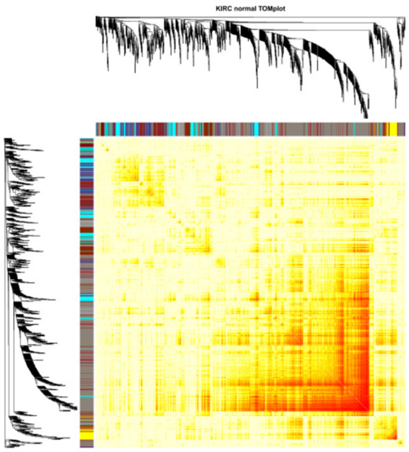

J

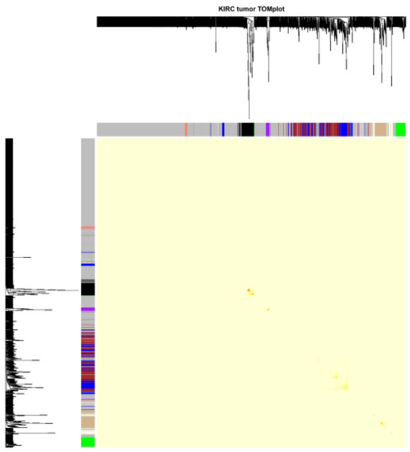

K

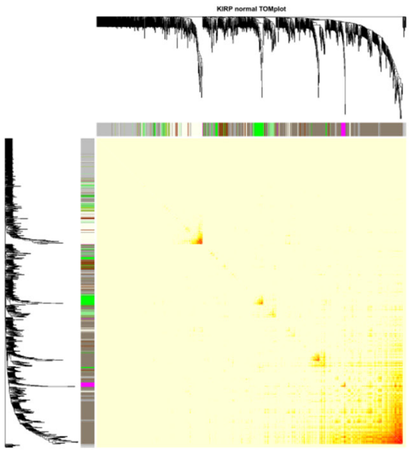

L

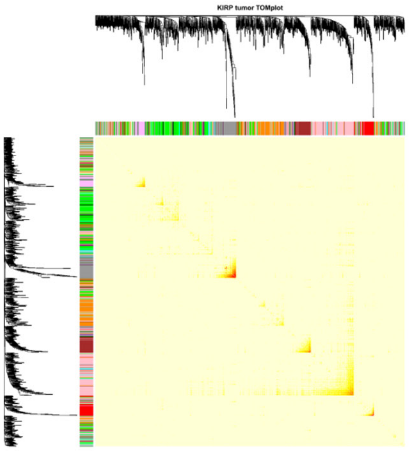

(*Continued*)

M

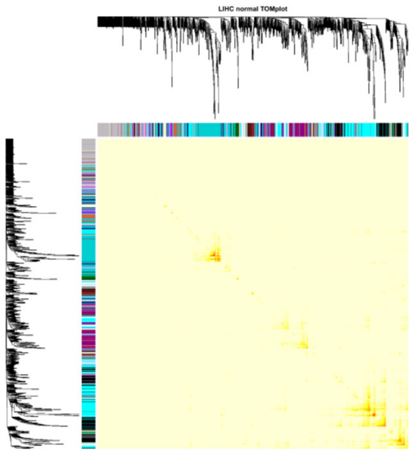

N

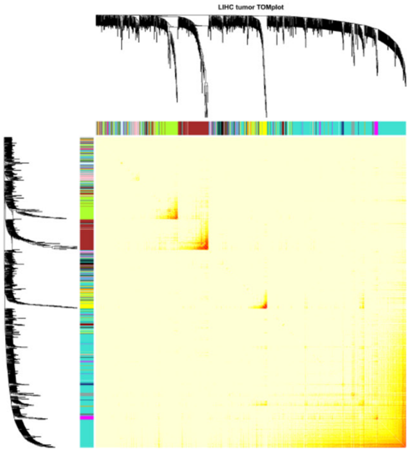

O

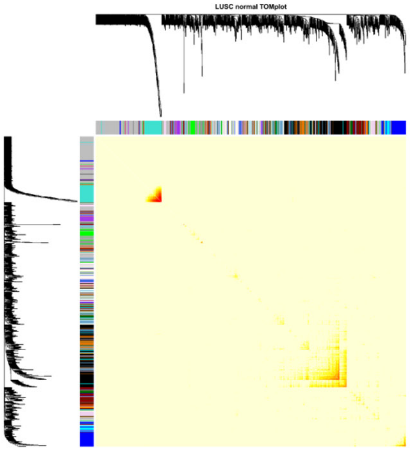

P

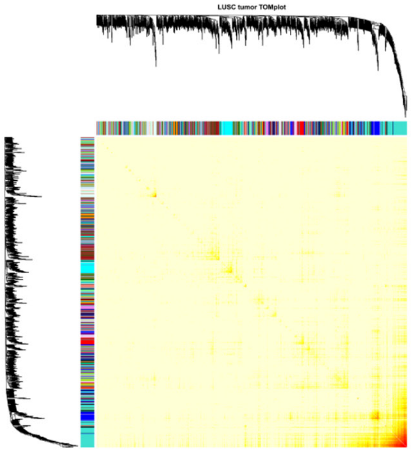

Q

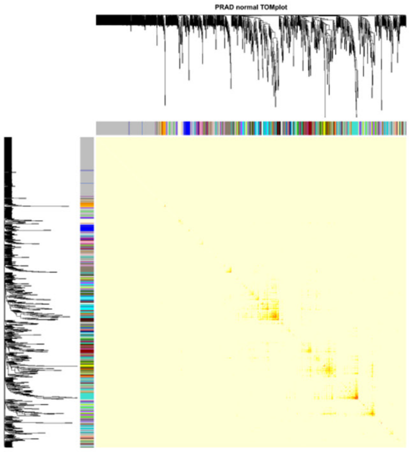

R

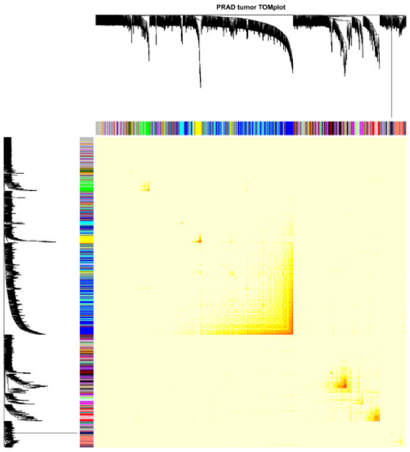

(Continued)

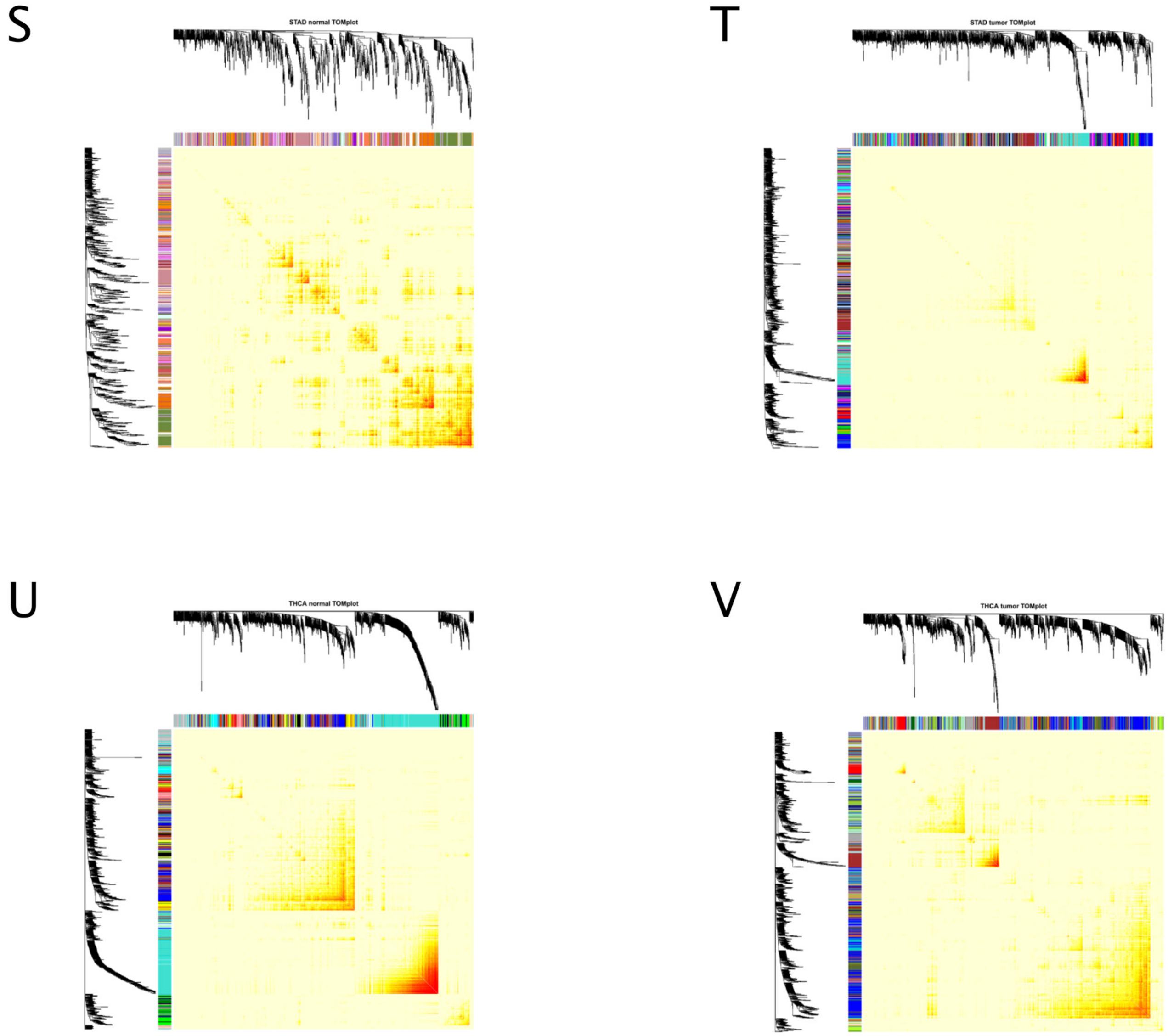

Supplementary Figure 4: Visualization of gene networks between normal tissues and corresponding tumors across 11 cancer types. Basically three patterns were observed, in CRC, KIRP, LUSC, and THCA, module structure was altered between normal tissue and cancer. And in BRCA, LIHC, and PRAD, more modules were found in cancers than in normal tissues. Last but not the least, it is worth noting that co-expressed gene modules were diminished or lost in HNSC, KICH, KIRC, LUAD, and STAD, in sharp contrast with the case in BRCA.

Supplementary Table 1: The TCGA samples used in this study.

See Supplementary File 1

Supplementary Table 2: Differentially expressed genes identified in this study.

See Supplementary File 2

Supplementary Table 3: Genes correlated with survival time in nine cancer types.

See Supplementary File 3
